# Intellectual and physical disability, household living arrangements and COVID-19 outcomes in Scotland: protocol for a retrospective cohort study

**DOI:** 10.1136/bmjopen-2026-117443

**Published:** 2026-06-23

**Authors:** Yusuff Adebayo Adebisi, Nick Bailey, Angela Henderson, Chris Dibben, Serena Pattaro

**Affiliations:** 1 Administrative Data Research Scotland, School of Social and Political Sciences, University of Glasgow, Glasgow, UK; 2 School of Health and Wellbeing, University of Glasgow, Glasgow, UK; 3 Administrative Data Research Scotland, School of Geosciences, University of Edinburgh, Edinburgh, UK

**Keywords:** COVID-19, Epidemiology, EPIDEMIOLOGY, Health Equity, PUBLIC HEALTH

## Abstract

**Abstract:**

**Introduction:**

While evidence consistently demonstrates elevated COVID-19 risks among people with intellectual and physical disabilities, less is known about how household living arrangements shape these inequalities. Residential setting and household size influence exposure, capacity to isolate and reliance on care yet have rarely been examined jointly with disability status at a population scale. This study aims to estimate disability-related inequalities in COVID-19 infection, hospitalisation and mortality in Scotland and to assess whether residential setting and household size modify these inequalities using linked, population-wide administrative data.

**Methods and analysis:**

This population-wide retrospective cohort study will use linked administrative data in Scotland. The study will focus on individuals present in the 2011 Scottish Census with records linkable to the Scottish population spine, which encompasses all individuals registered with a general practice who received a Community Health Index number. The cohort will comprise those who were alive, resident in Scotland and aged 16 years or older on 1 March 2020. Disability status will be classified from Census records as intellectual disability, physical disability (without intellectual disability) or a comparison group without reported disability. Address information recorded by the general practice will be linked to Ordnance Survey AddressBase Premium to assign Unique Property Reference Numbers. Residential settings will be classified from Basic Land and Property Unit codes as private dwellings or communal establishments and household size will be derived from resident counts at each property. Following linkage to the Public Health Scotland COVID-19 Research Database, outcomes including infection, hospitalisation and mortality will be ascertained from 1 March 2020 to 30 April 2022. Cox proportional hazards models will estimate associations between disability status, household living arrangements and COVID-19 outcomes, adjusting for relevant covariates. Effect modification will be assessed by evaluating interactions between disability status and household living arrangements on both multiplicative and additive scales.

**Ethics and dissemination:**

Ethical approval was granted by the College of Social Sciences Research Ethics Committee, University of Glasgow (reference: 400200099). Data linkage was approved by the Scottish Public Benefit and Privacy Panel for Health and Social Care and the Scottish Government & National Records of Scotland Data Access Panel (reference: 2021-0119). Findings will be disseminated through peer-reviewed open-access publications and conference presentations.

**STRENGTHS AND LIMITATIONS OF THIS STUDY**

- This study will use population-wide linked administrative data, enabling assessment of COVID-19 infection, hospitalisation and mortality across the adult population in Scotland.
- Linkage of Census, health and address-based data will allow classification of intellectual and physical disability alongside detailed residential setting and household size at a national scale.
- Derivation of household living arrangements using Unique Property Reference Numbers and Basic Land and Property Unit classifications will support examination of effect modification by private and communal residential settings.
- A key limitation is reliance on disability measures from the 2011 Census, which do not capture disability severity, support needs or changes in disability status by the start of the COVID-19 pandemic.
- Household living arrangements will be measured at baseline only, and ascertainment of SARS-CoV-2 infection will depend on recorded testing, which varied across pandemic waves.

## Introduction

 The COVID-19 pandemic represents both a public health emergency and a profound social crisis, exposing and amplifying deep-rooted structural inequalities across societies.^1 2^ Disabled individuals have been among the populations most severely affected in the UK and internationally.^3 4^ Globally, more than 1.3 billion people, approximately 16% of the world’s population, live with some form of disability, making disability one of the largest and most heterogeneous minority groups worldwide.^5^ In the UK, as of 2023/2024, an estimated 16.8 million people, nearly one in four of the population, report a long-term limiting disability,^6^ while in Scotland alone just over 1 million people (close to 20% of residents) report a limiting intellectual or physical disability.^7^ These figures underline the scale at which disability intersects with public health risk. The heightened risk of adverse outcomes among disabled people reflects biological susceptibility alongside entrenched social and economic disadvantages.^3 4^ The latter include reduced access to healthcare, lower health literacy, higher rates of poverty and deprivation and long-standing inequalities in the quality and timeliness of care, all of which shaped exposure and outcomes during the pandemic.^8 9^

Accumulating evidence demonstrates that disabled people face substantially higher risks of severe COVID-19 outcomes. A recent global systematic review and meta-analysis pooling data from 56 studies estimated that disabled people had a 2.7-fold higher risk of COVID-19-related death, rising to over threefold in population-based samples and being highest among those with intellectual disabilities.^10^ Multiple pathways may explain these inequalities. At the individual level, physical, intellectual and developmental impairments may limit the ability to adhere to preventive measures such as physical distancing, mask use or self-isolation.^11^ Many disabled individuals depend on close physical assistance from formal caregivers or family members, increasing contact intensity and exposure risk.^11^ The incidence of chronic health conditions such as diabetes, cardiovascular disease and respiratory illness is substantially higher among disabled populations,^12^,^14^ further increasing susceptibility to severe COVID-19 outcomes. In some conditions, biological mechanisms, including immune dysregulation, may also contribute to increased risk.^15^ Beyond these individual-level factors, systemic barriers within healthcare systems, including delayed access, communication barriers and discriminatory practices, have constrained timely diagnosis, treatment and vaccination.^16 17^ These vulnerabilities are further compounded by socioeconomic disadvantage, as disabled individuals are disproportionately represented among lower-income households and deprived neighbourhoods, where risks of exposure and severe disease are higher.^8 9^

Household living arrangements play a critical but underexplored role in shaping COVID-19 risks among disabled people. Congregate (group living) and residential care settings have been repeatedly identified as sites of intense transmission and excess mortality due to unavoidable close contact between multiple residents and caregivers.^18^,^20^ However, risks associated with household structure extend beyond institutional settings. Disabled individuals, particularly those with intellectual or developmental disabilities, are more likely to reside in larger households or shared living arrangements that facilitate informal care and support.^21^,^23^ While such arrangements can provide essential assistance, they also increase the number of close contacts and the likelihood of household transmission. Household socioeconomic conditions further modify these risks, as overcrowding, limited space for isolation and reduced access to resources may constrain the ability to implement infection prevention measures. These dynamics highlight the importance of examining disability within a household context rather than as an isolated individual characteristic.

Congregate living environments have consistently emerged as high-risk settings, with studies from Canada identifying nursing homes as among the most hazardous environments during the pandemic.^24^ Evidence from China has similarly demonstrated high transmission rates within households and healthcare facilities,^25^ while systematic reviews have documented elevated secondary transmission rates in indoor and shared community settings.^26^ Despite this, most studies examining disability and COVID-19 outcomes have focused on individual-level risk factors, with limited integration of household living arrangements. In England, analyses using the Office for National Statistics Public Health Data Asset demonstrated elevated COVID-19 mortality risks among disabled individuals, even after adjustment for household characteristics.^27^ However, reliance on 2011 Census data to define household units and under-ascertainment of intellectual disabilities in health records raise concerns about misclassification and residual bias. Consequently, the interaction between disability and household living arrangements remains insufficiently understood.

In Scotland specifically, the evidence base examining disability and COVID-19 outcomes remains limited. To date, only one population-level study has assessed COVID-19 outcomes among adults with intellectual disabilities using linked Census and health administrative data, reporting markedly elevated mortality ratios for both men and women.^28^ While this work provides important evidence of inequality, it does not consider physical disability, detailed household living arrangements, or the potential interaction between disability status and household context. This protocol describes a population-wide linked administrative data study examining these associations across the pandemic period in Scotland. This study forms part of a broader programme investigating social risk factors for COVID-19, established under the COVID-19 Data Intelligence Network and Data Taskforce.

### Aims and objectives

The overarching aim of this study is to leverage linked, population-wide administrative data in Scotland to examine how intellectual and physical disability, household living arrangements and their interaction shape the risk of adverse COVID-19 outcomes across the pandemic period.

Specifically, the objectives are:

1. To estimate disability-related differences in COVID-19 outcomes by comparing the risks of COVID-19 infection, hospitalisation and mortality among people with intellectual or physical disabilities with those among people without reported intellectual or physical disabilities.
2. To examine household-level determinants of COVID-19 outcomes by assessing the association between household living arrangements, including residential setting (private vs communal setting) and household size, and the risk of COVID-19 infection, hospitalisation and mortality.
3. To assess effect modification by household living arrangements by formally testing statistical interaction between disability status (intellectual and physical) and residential setting in relation to COVID-19 outcomes.

## Methods and analysis

### Study design and data sources

This study will be a retrospective cohort study using linked, routinely collected administrative data covering the majority of the Scottish population. Data linkage and analysis will be undertaken within Scotland’s national secure data environment, in accordance with established information governance, data protection and privacy requirements.^29^

Multiple national data sources will be linked for this study. The 2011 Scottish Census will provide baseline sociodemographic characteristics and self-reported intellectual and physical disability status. The Community Health Index (CHI) register will be used as the population spine to identify individuals registered with a Scottish general practice and to ascertain residential addresses at the start of the pandemic. Address fields extracted from current CHI records for individuals alive and resident in Scotland, or who died after 1 January 2020, yielded approximately 3.2 million unique address strings corresponding to around 5.8 million individuals, providing near-complete population coverage.^30^ These address records will be linked to Ordnance Survey AddressBase Premium to assign a Unique Property Reference Number (UPRN) to each residential location, enabling the derivation of household structure and classification of household living arrangements.^30^ Previous linkage exercises using this infrastructure have demonstrated that approximately 90% of CHI records can be successfully matched to a valid residential UPRN, supporting population-wide construction of household context.^30^ The remaining unmatched records may arise from incomplete or ambiguous address information, recent moves or non-standard address formats. Individuals without a valid residential UPRN linkage at the index date will be excluded from the analytic cohort. COVID-19 outcomes, including laboratory-confirmed infections, hospital admissions, vaccination records and deaths, will be obtained from the Public Health Scotland (PHS) COVID-19 Research Database.^31^

### Cohort construction and eligibility

**Figure 1:**
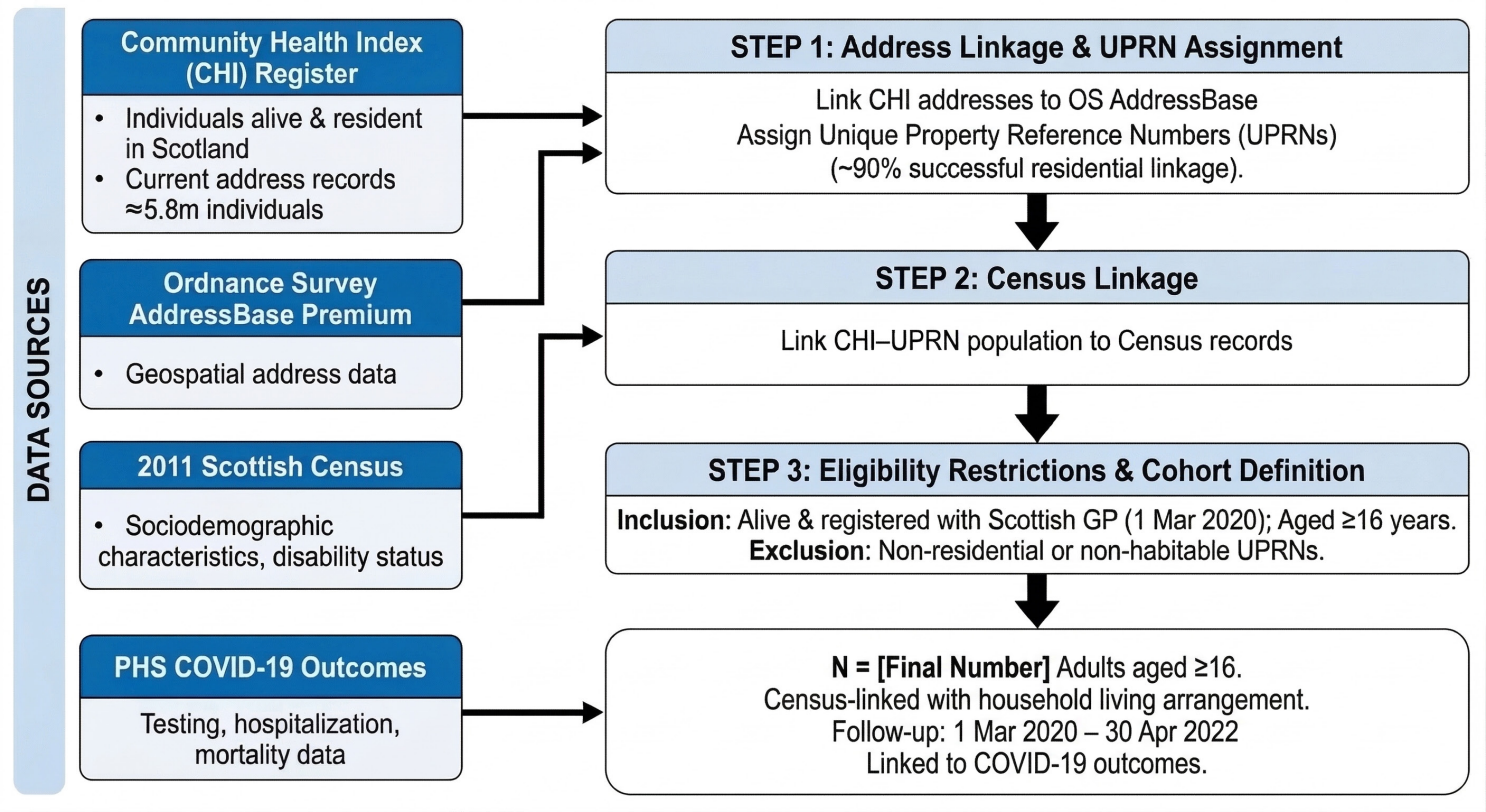
Planned data linkage and cohort construction. The figure illustrates the linkage of the Community Health Index (CHI) population spine to Ordnance Survey AddressBase Premium to assign Unique Property Reference Numbers (UPRNs), subsequent linkage to the 2011 Scottish Census and application of eligibility criteria to derive the analytic cohort of adults aged 16 years or older, with follow-up through linkage to Public Health Scotland COVID-19 outcome data. PHS, Public Health Scotland . OS, Ordnance Survey. GP, General Practitioner.

The study population will include individuals enumerated in the 2011 Scottish Census who were alive and registered with a Scottish general practice on 1 March 2020 (index date), corresponding to the effective start of the pandemic in Scotland.^32^ Eligibility will be restricted to individuals aged 16 years or older at baseline to support more reliable disability ascertainment and to reflect meaningful variation in living arrangements. Cohort members will be identified by linking Census records to the CHI register; individuals who died before the index date will be excluded as will those not present in the 2011 Census. To minimise exposure misclassification, individuals, without a valid residential UPRN at the index date or those linked to non-residential or non-habitable property types, will be excluded. Follow-up will extend from 1 March 2020 to 30 April 2022. A flow diagram illustrating the planned data linkage steps, eligibility criteria and derivation of the final analytic cohort is shown in figure 1.

### Disability status

Disability status will be derived from responses to question 18 of the 2011 Scottish Census,^33^ which asked respondents: “Do you have any of the following which have lasted or are expected to last at least 12 months?*”* Respondents could select one or more categories, including sensory impairments, physical disability, learning disability, learning difficulty, developmental disorder, mental health conditions, long-term illness, other conditions or no condition.^33^ Based on these responses, individuals will be classified into three mutually exclusive groups using a sequential approach:

1. Individuals who reported learning disability will be classified as having an intellectual disability, regardless of any additional conditions reported. In the Scottish Census context, learning disability denotes a lifelong condition with onset in the developmental period that affects intellectual functioning, adaptive behaviour and learning and corresponds to the WHO International Classification of Diseases, 11th Revision (ICD-11) definition of disorders of intellectual development.^34^ For clarity, we refer to the Census category ‘learning disability’ as intellectual disability throughout this paper.
2. Individuals who did not report intellectual disability but reported physical disability and/or sensory impairments (including deafness or partial hearing loss and blindness or partial sight loss) will be classified as having a physical disability. This group captures chronic motor and sensory impairments that may influence exposure risk, mobility, access to healthcare and indicate potential reliance on household or institutional support.^35^
3. Individuals will be classified into a comparison group if they are not included in either the intellectual disability or physical disability groups. This group therefore comprises people who did not report a learning disability (treated as intellectual disability in this study) and did not report a long-term condition substantially limiting day-to-day activities (treated as physical disability), including those who reported no condition and those reporting other Census categories (eg, mental health conditions, long-term illness, learning difficulty, developmental disorder or other conditions). The comparison group is intended to represent individuals without reported intellectual or physical disability rather than the absence of health conditions more broadly. These additional categories are heterogeneous and may capture distinct constructs and pathways (including potential links with congregate living) that are outside the scope of this analysis; we therefore do not interpret the comparison group as ‘healthy’ and note this as a limitation and priority for future work.

The use of disability status derived from the 2011 Census introduces a temporal gap of approximately 9 years before the start of the pandemic observation period. For intellectual disability, the risk of misclassification is considered relatively low, as intellectual disabilities are lifelong conditions with onset in the developmental period, and the cohort was restricted to individuals aged 16 years or older at baseline, by which point identification is expected to be largely complete. For physical disability, the measurement gap is more consequential. Although the Census question emphasises durability of condition (‘have lasted or are expected to last at least 12 months’) and underwent cognitive testing to support reliable reporting, the physical disability cohort is expected to be substantially older on average, reflecting increased disability prevalence with age. Consequently, a proportion of individuals in the comparison group will have acquired physical disabilities during the intervening period, which the 2011 Census measure cannot capture. This form of non-differential misclassification is expected to attenuate observed associations between physical disability and COVID-19 outcomes, biasing estimates towards the null. The potential impact of this measurement gap on observed associations for physical disability will be explicitly acknowledged when interpreting the findings.

### Household living arrangements

Household living arrangements will be derived using UPRN-based linkage rather than self-reported survey data. Individuals sharing the same UPRN at the index date will be considered to reside in the same household or residential setting.^30^ This approach enables population-wide, reproducible classification of living arrangements across Scotland.

Residential setting will be classified using the Basic Land and Property Unit (BLPU) codes attached to each UPRN. A BLPU is the basic unit used in address databases in Great Britain, and each BLPU with a UPRN is assigned a classification that identifies its use and type, including residential dwelling categories.^36^ Two broad categories will be defined: private dwellings and communal establishments. Private dwellings will include residential houses, flats, maisonettes, sheltered housing and houses in multiple occupation. Communal establishments will include residential institutions such as care homes, nursing homes, residential schools, halls of residence and hostels. They will be further split using the UPRN-linked resident count as small (<50 linked residents) and large (≥50 linked residents) establishments. Non-habitable property types will be excluded.

Household size will be derived by counting all CHI-registered residents at the same UPRN at the index date,^37 38^ including children; as noted above, analytic models only include individuals aged 16 years or older. Household size will be categorised into five groups: 1 resident, 2–4 residents, 5–7 residents, 8–10 residents and 11 or more residents. Household size has been used as an important contextual factor in COVID-19 epidemiology, with prior studies operationalising categories such as single occupancy, two-person households and households with three or more residents.^39^ The more granular categorisation adopted in this study is intended to capture increasing levels of residential density and potential exposure to within-household transmission.

Where necessary, sensitivity analyses will assess the robustness of findings to alternative household size groupings based on the observed distribution in the analytic cohort.

### Outcomes

The primary outcomes of interest will be COVID-19 infection, hospitalisation and mortality. COVID-19 infection will be defined as a laboratory-confirmed positive SARS-CoV-2 test recorded in the PHS COVID-19 Research Database during the follow-up period.^40^ Hospitalisation will be defined as any inpatient admission with COVID-19 recorded as a primary or secondary diagnosis or an admission occurring within 14 days of a confirmed positive test.^40^ Mortality will be defined as death with COVID-19 recorded as a primary or secondary cause on the death certificate or occurring within 28 days of a confirmed infection, consistent with national surveillance definitions.^40^

### Covariates

**Figure 2:**
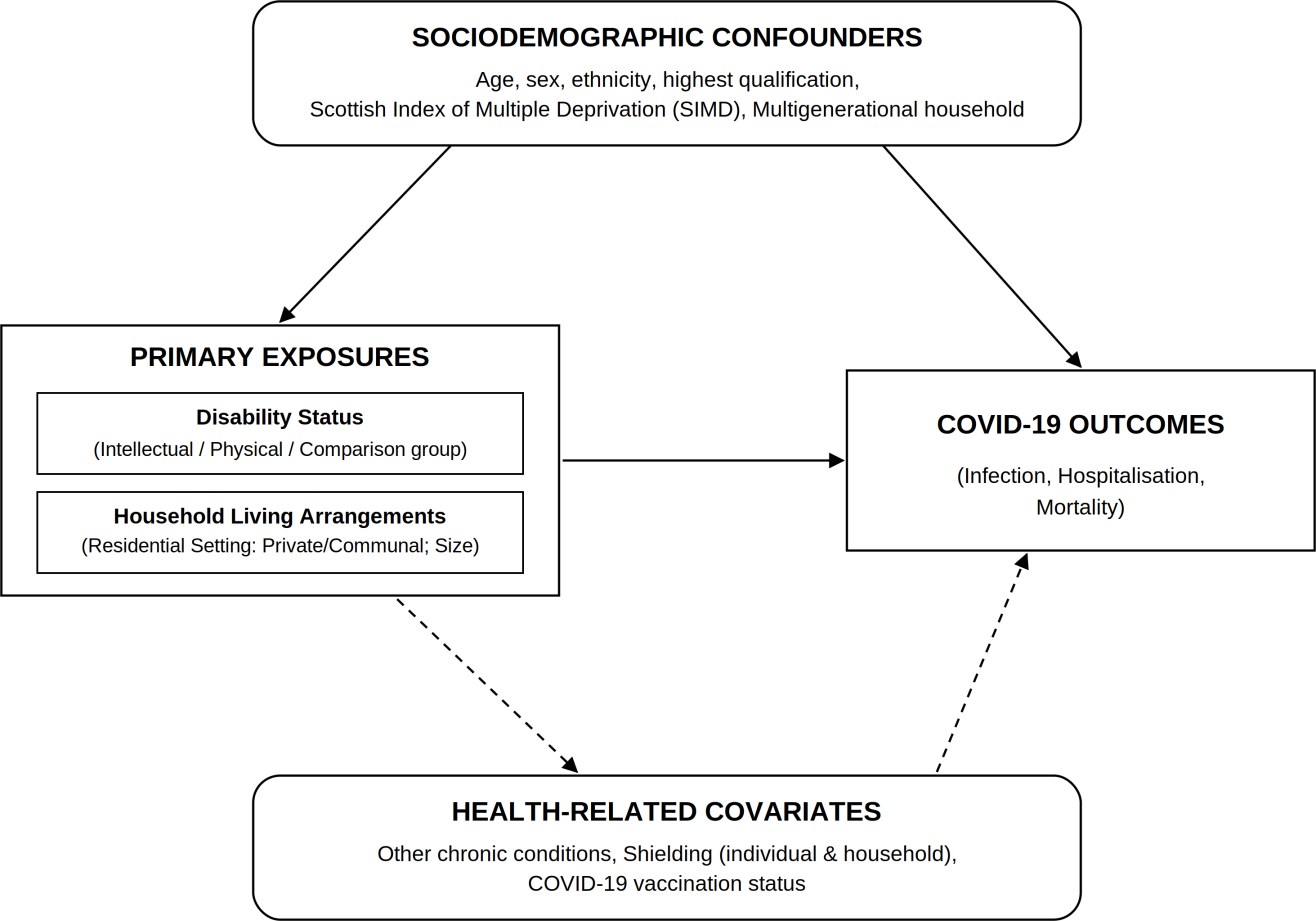
Directed acyclic graph of the hypothesised relationships between disability status, household living arrangements and COVID-19 outcomes. Disability status (intellectual, physical or comparison group) and household living arrangements (residential setting and household size) are the primary exposures. Sociodemographic factors (age, sex, ethnicity, highest qualification, area-level deprivation (Scottish Index of Multiple Deprivation (SIMD)) and multigenerational household status) will be treated as confounders and adjusted for in multivariable models. Health-related covariates (other chronic conditions, shielding and COVID-19 vaccination status) may lie on the causal pathway from disability status to COVID-19 outcomes, as indicated by the dashed arrow, and will therefore be introduced in later stages of the sequential adjustment strategy used for the disability–outcome analyses.

A set of individual-level and household-contextual covariates will be included to characterise the cohort and support adjustment of planned analyses. Covariates were selected a priori based on established evidence of relevance to COVID-19 infection and severity as well as availability within the linked administrative datasets. These covariates will allow adjustment for key demographic, socioeconomic, clinical and household-contextual factors that may confound observed associations between disability status, household living arrangements and COVID-19 outcomes (see figure 2).

Individual-level sociodemographic covariates will include age, sex, ethnicity, educational attainment and area-level socioeconomic deprivation. Age will be derived from the CHI register and modelled as a continuous variable (in years) and, where appropriate, in categories (16–34, 35–49, 50–64, 65 or more years). Sex will be obtained from CHI and categorised as male or female. Ethnicity will be derived from the 2011 Scottish Census and grouped as white versus non-white to preserve statistical power and treated as a social indicator. Educational attainment, obtained from the Census, will be included as an indicator of socioeconomic position and health literacy. Area-level deprivation will be measured using the Scottish Index of Multiple Deprivation (SIMD) 2020, categorised into quintiles. The SIMD is a composite measure that orders small geographical areas with comparable population sizes according to relative deprivation across seven standardised domains (income, employment, education, health, access to services, crime and housing) and is widely used to guide policies and interventions aimed at reducing social inequalities.^41^

Health-related covariates will include the presence of other long-term health conditions, individual shielding status and COVID-19 vaccination status. Long-term illness or health conditions will be derived from the Census and coded as a binary indicator capturing conditions other than the primary disability exposure. Individual shielding status will be identified from the Public Health Scotland Shielding Patient List and included as a marker of clinical vulnerability and behavioural risk modification.^42^ COVID-19 vaccination status will be obtained from the PHS COVID-19 Research Database and categorised according to the number of doses received (zero, one, two or three or more) and will be treated as baseline or time-varying where feasible.

Household-contextual covariates will include multigenerational household status and household-level shielding exposure on the basis that both could affect the behaviour of household members. Multigenerational household status will be derived from UPRN-based household identifiers and defined as UPRN-linked households containing at least one person aged≥65 years and at least one co-resident who is≥20 years younger (based on ages at the March 2020 index date). Household-level shielding exposure will be defined as the presence of at least one other household member on the shielding patient list, capturing indirect exposure to shielding behaviours within the household environment.

### Planned statistical analysis

All analyses will be conducted within the Scottish National Safe Haven using approved statistical software, in accordance with data governance and disclosure control requirements.^29^ The statistical analysis plan is pre-specified and structured to correspond directly to the three primary study objectives.

Baseline characteristics of the cohort will be summarised descriptively and stratified by disability status and household living arrangements. Continuous variables will be presented as means and SD or medians and IQRs, as appropriate, and categorical variables as counts and percentages.

Cox proportional hazards regression will be the primary modelling framework for time-to-event analysis of COVID-19 outcomes. Time at risk will be defined from study onset (1 March 2020) until the first occurrence of the outcome of interest, death or end of follow-up (30 April 2022), whichever occurs first. Separate models will be fitted for each outcome (infection, hospitalisation and mortality). HRs with 95% CIs will be reported for all models. All Cox proportional hazards models will account for within-household clustering by applying robust standard errors at the UPRN level.

For the first objective, disability-related differences in COVID-19 outcomes will be estimated using Cox proportional hazards models with disability status (intellectual disability, physical disability and comparison group) as the primary exposure. Sequential models will be fitted to examine the contribution of successive covariate domains: Model 1 will be unadjusted; Model 2 will adjust for demographic characteristics (age and sex); Model 3 will additionally adjust for socioeconomic and ethnic factors (ethnicity, area-level deprivation, highest educational qualification and multigenerational household status); Model 4 will further adjust for individual- and household-level shielding status; Model 5 will additionally adjust for the presence of other chronic health conditions and Model 6 will additionally adjust for COVID-19 vaccination status. This sequential approach allows examination of how estimated associations attenuate as successive covariate domains are introduced, providing insight into the pathways through which disability-related inequalities may operate. Variables such as shielding status and vaccination status may function partly as mediators on the causal pathway between disability and COVID-19 outcomes; their inclusion in later models is intended to assess the extent to which these factors account for observed associations, and differences across model stages will be interpreted accordingly.

For the second objective, household-level determinants of COVID-19 outcomes will be examined using Cox proportional hazards models with household living arrangements as the primary exposures, including residential setting and household size categories. Unadjusted models will be fitted first, followed by fully adjusted models including age, sex, ethnicity, area-level deprivation, highest educational qualification, individual and household-level shielding status, other chronic health conditions, disability status and COVID-19 vaccination status, to estimate the independent associations between household living arrangements and COVID-19 infection, hospitalisation and mortality. Multigenerational household status will not be included in these models, as it is a household compositional characteristic that is intrinsically related to the primary exposures of residential setting and household size; adjusting for it could attenuate the very associations under investigation.

For the third objective, effect modification by household living arrangements will be evaluated on both multiplicative and additive scales. Multiplicative interaction will be assessed by including product terms between disability status (intellectual and physical) and household living arrangements (residential setting and household size) in Cox regression models fully adjusted for age, sex, ethnicity, area-level deprivation, highest educational qualification, individual shielding, household-level shielding, other chronic health conditions and vaccination status, with statistical significance evaluated using likelihood ratio tests comparing models with and without interaction terms. Additive interaction will be assessed by calculating the relative excess risk due to interaction (RERI) with 95% CIs, as additive departures from expected joint effects are considered more relevant for identifying groups at disproportionate risk and for informing public health intervention. Where evidence of interaction is observed on either scale, stratum-specific HRs will be presented to facilitate interpretation. In practical terms, multiplicative interaction indicates whether the combined effect of disability and a given household living arrangement exceeds the product of their separate relative effects, whereas additive interaction indicates whether their combined absolute effect exceeds the sum of their separate effects. Both scales provide complementary information: multiplicative interaction is the conventional measure reported in epidemiological studies, while additive interaction is particularly informative for public health planning, as it identifies subgroups in which disability and household context together produce disproportionately elevated absolute risk and therefore where targeted interventions may have the greatest impact.

The proportional hazards assumption will be assessed using Schoenfeld residuals and graphical diagnostics using log-log plots. If substantial departures from proportionality are identified, alternative model specifications (eg, stratified Cox models or inclusion of time-varying coefficients) will be considered. Death from non-COVID-19 causes may act as a competing event for infection and hospitalisation outcomes. Cause-specific Cox proportional hazards models will be used throughout rather than subdistribution (Fine-Gray) approaches, consistent with the aetiological aim of quantifying how disability and household living arrangements influence the rate at which COVID-19 events occur, rather than predicting cumulative incidence in the presence of competing events. The potential influence of competing risks on the interpretation of cumulative measures will be acknowledged as a limitation. Given the number of planned comparisons across three outcomes, two exposure dimensions and interaction analyses, results will be interpreted with attention to the pattern and consistency of findings rather than reliance on individual p-values. Formal adjustment for multiple testing (eg, Bonferroni correction) will not be applied, as the analyses are pre-specified and hypothesis-driven, but the potential for inflated type I error will be acknowledged.

All analyses will be reported in accordance with the REporting of studies Conducted using Observational Routinely-collected Data (RECORD) and Strengthening the Reporting of Observational Studies in Epidemiology (STROBE) guidelines for studies using routinely collected health data.^43^

### Patient and public involvement

Patient and public involvement was incorporated at the design stage of this research through engagement with the Scotland Talks Data Public Panel, a forum representing diverse public interests and lived experiences. Feedback was sought during a presentation of the project concept in April 2024, which focused on household living arrangements, disability and COVID-19 outcomes in Scotland. Panel members, including individuals with lived experience of disability during the COVID-19 pandemic, expressed strong support for the research and highlighted the importance of understanding why disabled people experienced disproportionate impacts and how such inequalities might be mitigated in future public health emergencies.^44^

The panel provided input on the perceived benefits of the research, potential concerns and priorities for dissemination. Members emphasised that disabled people were often left behind during the pandemic and relied heavily on third-sector and community support, reinforcing the relevance and urgency of the study. The panel also supported the value of using linked residential data to address gaps in existing evidence and highlighted the importance of learning lessons to inform future pandemic preparedness. Feedback further underlined interest in extending the work into a longer-term research programme, including potential linkage with future Census data, while noting the importance of sustainable resourcing and governance.^44^

## Ethics and dissemination

###  Ethical approval and data governance

This study will use linked, routinely collected administrative data and will be conducted within Scotland’s national secure data environment. All data linkage, storage and analysis will take place within the Scottish National Safe Haven in accordance with national information governance, data protection and confidentiality requirements, including compliance with the UK General Data Protection Regulation (GDPR) and the Data Protection Act 2018.^29^ Data will be pseudonymised prior to researcher access, and linkage will be undertaken by Electronic Data Research and Innovation Service (eDRIS). Researchers will access only pseudonymised analytical datasets, and all outputs will be subject to statistical disclosure control prior to release.

Ethical approval for the study was granted by the College of Social Sciences Research Ethics Committee, University of Glasgow (reference: 400200099). Approval for data linkage and use of the data for research purposes was obtained from the Scottish Public Benefit and Privacy Panel for Health and Social Care and the Scottish Government & National Records of Scotland Data Access Panel (reference: 2021-0119).

### Dissemination plan

Findings from this study will be disseminated through peer-reviewed open-access journal publications and presentations at national and international academic conferences. In addition to academic dissemination, the research team will engage with policymakers, public health agencies and third-sector organisations to support translation of findings into policy and practice.

## Discussion

This research has important implications for public health policy by highlighting disability and household living arrangements as intersecting determinants of vulnerability during infectious disease emergencies. By generating population-wide evidence on how intellectual and physical disability interact with residential setting and size to shape COVID-19 risks, the study may contribute evidence relevant to more targeted and equitable preparedness strategies. In particular, findings could inform future discussions on risk stratification frameworks used for pandemic planning, including vaccination prioritisation, shielding guidance and allocation of community-based support. Incorporating household context alongside individual vulnerability could enable policymakers to better identify groups at heightened risk who may be overlooked by approaches focused solely on age or specific clinical conditions. Such evidence is especially relevant for improving responses in communal and high-density living settings, where infection control measures must balance protection with residents’ autonomy and care needs. More broadly, the study reinforces the importance of routinely linking social and residential data with health records to support timely, equity-focused decision-making in future public health crises.^45 46^

The findings may also inform the design and delivery of services across health, social care and third-sector organisations supporting disabled people. Improved understanding of how household context shapes exposure and outcomes can guide tailored interventions, such as targeted outreach, accessible public health messaging and enhanced in-home or community-based support during periods of heightened risk. Evidence from this study could also support closer integration between public health surveillance and social care planning, particularly in identifying households where disabled individuals may require additional resources to safely isolate or access care. In the longer term, the research provides a framework for embedding disability and residential context into routine health monitoring and emergency response planning, helping ensure that future interventions are proportionate, inclusive and responsive to the lived realities of disabled populations.

This study has several anticipated strengths and limitations that should be considered when interpreting the eventual findings. A major strength is the use of population-wide linked administrative data, which will enable a comprehensive assessment of COVID-19 infection, hospitalisation and mortality across virtually all adults in Scotland, while reducing selection bias inherent in survey-based studies. The novel linkage of Census, health and address-based records will allow the joint examination of intellectual and physical disability alongside detailed residential context, an approach rarely achieved at this scale. The derivation of household living arrangements from UPRN-based linkage will provide an objective, reproducible classification that is independent of self-report.

However, the study will also have important limitations. Disability status will be derived from the 2011 Census, introducing a 9-year measurement gap before the pandemic. For intellectual disability, the risk of misclassification is likely to be lower because of its lifelong nature and developmental onset. In contrast, for physical disability, age-related acquisition of disability during the intervening period means that some individuals classified as non-disabled in 2011 may have become disabled by 2020. This would likely attenuate observed associations. The Census measure does not capture disability severity, support needs or functional limitations, which may further limit the precision of exposure classification. UPRN-based household linkage, while innovative, does not capture within-household dynamics such as caregiving intensity or contact patterns, and approximately 10% of records cannot be matched to a valid UPRN, introducing potential selection bias. Ascertainment of COVID-19 infection will depend on recorded testing, which varied substantially across pandemic waves, potentially leading to differential outcome misclassification. Household living arrangements will be measured at a single time point and may not reflect changes in living situation during the follow-up period. Finally, residual confounding from unmeasured factors, including behavioural responses to public health guidance and access to informal care, cannot be excluded.
