# Supplementary material for: Intellectual and physical disability, household living arrangements and COVID-19 outcomes in Scotland: protocol for a retrospective cohort study
Source: BMJ Open. 2026 Jun 23;16(6):e117443. doi: 10.1136/bmjopen-2026-117443 (PMC13295980; doi:10.1136/bmjopen-2026-117443)
Supplement: Supplementary file 1 [file bmjopen-2026-117443.reviewer_comments.pdf]

PEER REVIEW HISTORY

BMJ Open publishes all reviews undertaken for accepted manuscripts. Reviewers are asked to complete a checklist review form and are provided with free text boxes to elaborate on their assessment. These free text comments are reproduced below.

ARTICLE DETAILS

Title (Provisional)

Intellectual and physical disability, household living arrangements, and COVID-19 outcomes in Scotland: protocol for a retrospective cohort study

Authors

Adebisi, Yusuff Adebayo; Bailey, Nick; Henderson, Angela; Dibben, Chris; Pattaro, Serena

---

VERSION 1 - REVIEW

**Reviewer**	**1**
**Name**	**Fernández-Lázaro, Diego**
**Affiliation**	**Universidad de Valladolid Facultad de Ciencias**
**Date**	**25-Mar-2026**
**COI**	**None**

Thank you for the opportunity to review your manuscript entitled "Intellectual and Physical Disability, Household Living Arrangements, and COVID-19 Outcomes in Scotland: A Retrospective Cohort Study Protocol" .

General assessment

This protocol addresses an important and timely research question in social epidemiology: how disability status and household living arrangements jointly shape COVID-19 outcomes. The use of population-wide linked administrative data is a major strength and gives the study strong potential to contribute to public health policy and inequalities research.

Overall, the protocol is well structured and broadly aligned with RECORD/STROBE guidance. However, several methodological aspects require clarification and further discussion before the manuscript can be considered for publication.

Major comments

1. Definition and measurement of disability

Disability status is derived from the 2011 Census, which introduces a substantial temporal gap (~9 years before the pandemic).

This raises concerns regarding:

Potential misclassification due to changes in disability status over time

Lack of information on severity, functional limitations, or care needs

I recommend expanding the discussion on:

Possible misclassification bias (differential vs non-differential)

Implications for interpretation of results

Potential sensitivity analyses (if feasible)

2. Measurement of household living arrangements

The use of UPRN-based linkage is innovative and a clear strength.

However, it does not capture:

Within-household dynamics (e.g., caregiving roles, contact intensity)

Potential inaccuracies in address records

The data linkage process (Figure 1, page 7) is well presented, but the manuscript should:

Clarify expected linkage errors (~90% success rate is mentioned)

Explain how unmatched or uncertain cases will be handled

3. Confounding and causal framework

The DAG (Figure 2, page 10) is a strength and demonstrates conceptual clarity.

However:

The criteria for covariate selection are not fully explicit

Some variables (e.g., shielding status, vaccination) may act as mediators rather than confounders

Please clarify:

Whether the aim is to estimate total or direct effects

The rationale for adjustment strategy in relation to the DAG

4. Statistical analysis plan

The use of Cox proportional hazards models is appropriate.

However:

There is no discussion of competing risks, particularly for mortality outcomes

Interaction analyses may require substantial statistical power

Consider:

Addressing competing risks (e.g., Fine–Gray models) or justifying their exclusion

Clarifying approach to multiple testing and model complexity

5. Potential sources of bias

While some limitations are acknowledged, the discussion should be expanded to include:

Selection bias:

Exclusion of individuals without valid UPRN linkage

Information bias:

Dependence on recorded SARS-CoV-2 testing (which varied across time)

Temporal bias:

Changes across pandemic waves (testing, vaccination, variants)

6. Scope and interpretation

Some sections (particularly implications) appear slightly anticipatory for a protocol.

As per protocol reporting standards, conclusions should remain strictly focused on planned analyses rather than expected findings.

Minor comments

The introduction is comprehensive but could be more concise.

Some methodological sections could be streamlined for clarity.

Ensure all abbreviations (e.g., UPRN, BLPU) are clearly defined at first use.

Figures (especially Figure 2) could be improved in readability.

Strengths

Population-wide linked administrative data

Novel focus on interaction between disability and household context

Strong conceptual framework (including DAG)

High relevance for public health and policy

Conclusion

This is a well-conceived and potentially impactful protocol. However, important clarifications are needed regarding:

- Exposure definitions

- Analytical strategy

VERSION 1 - AUTHOR RESPONSE

Reviewer 1 Comments

General assessment

> **Comment:** *This protocol addresses an important and timely research question in social epidemiology: how disability status and household living arrangements jointly shape COVID-19*

> *outcomes. The use of population-wide linked administrative data is a major strength and gives the study strong potential to contribute to public health policy and inequalities research. Overall, the protocol is well structured and broadly aligned with RECORD/STROBE guidance. However, several methodological aspects require clarification and further discussion before the manuscript can be considered for publication.*

Response: We thank the reviewer for this positive assessment and for the detailed and constructive feedback. We address each of the points raised below.

Major Comments

1. Definition and measurement of disability

> **Comment:** *Disability status is derived from the 2011 Census, which introduces a substantial temporal gap (~9 years before the pandemic). This raises concerns regarding: Potential misclassification due to changes in disability status over time; Lack of information on severity, functional limitations, or care needs. I recommend expanding the discussion on: Possible misclassification bias (differential vs non-differential); Implications for interpretation of results; Potential sensitivity analyses (if feasible).*

Response: We have substantially expanded the discussion of the disability measurement gap. A new paragraph has been added after the exposure definitions (Disability status section) that addresses this issue in detail. For intellectual disability, we argue that the risk of misclassification is relatively low, as intellectual disabilities are lifelong conditions with onset in the developmental period, and the cohort was restricted to individuals aged 16 years or older at baseline, by which point identification is expected to be largely complete. This argument is supported by reference to the WHO ICD-11 definition of disorders of intellectual development (new reference 34). The reference to the Census definition has been updated to specify the "Scottish Census context" to differentiate from the English/Welsh Census approach. For physical disability, we acknowledge that the measurement gap is more consequential, as the onset of physical disability has a clear relationship with age, and a proportion of individuals categorised in the comparison group will have acquired disabilities during the intervening period. We characterise this as non-differential misclassification expected to attenuate observed associations, biasing estimates towards the null. Sensitivity analyses to assess the potential impact of this misclassification are noted. These limitations are further discussed in the new strengths and limitations paragraph within the Discussion section.

2. Measurement of household living arrangements

> **Comment:** *The use of UPRN-based linkage is innovative and a clear strength. However, it does not capture: Within-household dynamics (e.g., caregiving roles, contact intensity); Potential inaccuracies in address records. The data linkage process (Figure 1, page 7) is well presented, but the manuscript should: Clarify expected linkage errors (~90% success rate is mentioned); Explain how unmatched or uncertain cases will be handled.*

Response: We have added clarification in the Study design and data sources section following the mention of the approximately 90% linkage rate. The text now explains that the remaining unmatched records may arise from incomplete or ambiguous address information, recent moves, or non-standard address formats, and that individuals without a valid residential UPRN linkage at the index date will be excluded from the analytic cohort. The limitations of UPRN-based linkage, including the inability to capture within-household dynamics such as caregiving intensity or contact patterns, and the

potential for selection bias arising from approximately 10% of unmatched records, are discussed in the new strengths and limitations paragraph within the Discussion section. Figure 1 has been revised for improved clarity.

3. Confounding and causal framework

> **Comment:** *The DAG (Figure 2, page 10) is a strength and demonstrates conceptual clarity. However: The criteria for covariate selection are not fully explicit; Some variables (e.g., shielding status, vaccination) may act as mediators rather than confounders. Please clarify: Whether the aim is to estimate total or direct effects; The rationale for adjustment strategy in relation to the DAG.*

Response: We thank the reviewer for this important comment. Several changes have been made to address it comprehensively. First, Figure 2 has been revised to distinguish confounding and mediating pathways: solid arrows now represent assumed causal relationships, while dashed arrows indicate potential mediating pathways through which exposures may influence COVID-19 outcomes indirectly via health-related factors. The figure legend has been updated accordingly, and the label "Health & Risk Confounders" has been changed to "Health-related covariates" to avoid implying that all variables in this box are confounders. Second, the Planned statistical analysis section now explicitly states that the primary analytic aim is to estimate the total effect of disability status on COVID-19 outcomes. Covariates were selected a priori based on the DAG. The sequential adjustment strategy (Models 1–6) is designed to examine how estimated associations attenuate as successive covariate domains are introduced. Variables such as shielding status and vaccination status, which may function partly as mediators on the causal pathway between disability and COVID-19 outcomes, are introduced in later model stages (Models 4 and 6) to assess the extent to which they account for observed associations, rather than being included from the outset where they could block mediating pathways. Differences across model stages will be interpreted accordingly.

4. Statistical analysis plan

> **Comment:** *The use of Cox proportional hazards models is appropriate. However: There is no discussion of competing risks, particularly for mortality outcomes; Interaction analyses may require substantial statistical power. Consider: Addressing competing risks (e.g., Fine–Gray models) or justifying their exclusion; Clarifying approach to multiple testing and model complexity.*

Response: We have added a detailed discussion of competing risks to the Planned statistical analysis section. Because the primary analytic interest is in estimating the cause-specific hazard of each COVID-19 outcome — that is, the instantaneous rate of the event among individuals who have not yet experienced it — cause-specific Cox proportional hazards models are used throughout rather than subdistribution (Fine–Gray) approaches. This is consistent with the aetiological focus of the study, where the objective is to quantify how disability and household living arrangements influence the rate at which COVID-19 events occur, rather than to predict cumulative incidence in the presence of competing events. The potential influence of competing risks on the interpretation of cumulative measures is acknowledged as a limitation. Regarding multiple testing, we have added text acknowledging that given the number of planned comparisons across three outcomes, two exposure dimensions, and interaction analyses, results will be interpreted with attention to the pattern and consistency of findings rather than reliance on individual p-values. Formal adjustment for multiple testing will not be applied, as the analyses are pre-specified and hypothesis-driven, but the potential for inflated type I error is acknowledged. Additionally, the interaction analysis section has been expanded to include both multiplicative interaction (assessed via likelihood ratio tests) and additive

interaction (assessed via the Relative Excess Risk due to Interaction, RERI, with 95% confidence intervals), as additive departures are considered more relevant for identifying groups at disproportionate risk.

5. Potential sources of bias

> **Comment:** *While some limitations are acknowledged, the discussion should be expanded to include: Selection bias: Exclusion of individuals without valid UPRN linkage; Information bias: Dependence on recorded SARS-CoV-2 testing (which varied across time); Temporal bias: Changes across pandemic waves (testing, vaccination, variants).*

Response: We have added a comprehensive strengths and limitations paragraph to the Discussion section that addresses all three sources of bias identified by the reviewer. Selection bias: we note that approximately 10% of records cannot be matched to a valid UPRN, introducing potential selection bias, and that individuals without valid UPRN linkage are excluded from the analytic cohort. Information bias: we acknowledge that ascertainment of COVID-19 infection depends on recorded testing, which varied substantially across pandemic waves, potentially leading to differential outcome misclassification. Temporal bias: we note that household living arrangements are measured at a single time point and may not reflect changes in living circumstances during the follow-up period. Residual confounding from unmeasured factors, including behavioural responses to public health guidance and access to informal care, is also acknowledged.

6. Scope and interpretation

> **Comment:** *Some sections (particularly implications) appear slightly anticipatory for a protocol. As per protocol reporting standards, conclusions should remain strictly focused on planned analyses rather than expected findings.*

Response: We agree with the reviewer. The Implications section has been renamed "Discussion" and the language has been softened throughout to be appropriately tentative. For example, "the study can inform more targeted and equitable preparedness strategies" has been changed to "the study may contribute evidence relevant to more targeted and equitable preparedness strategies," and "findings may support the refinement of risk stratification frameworks" has been changed to "findings could inform future discussions on risk stratification frameworks." All statements now refer to the potential contributions of the planned analyses rather than anticipated findings.

Minor Comments

> **Comment:** *The introduction is comprehensive but could be more concise.*

Response: The final paragraph of the introduction has been substantially trimmed. Material relating to data linkage specifics and disability measurement caveats has been removed from the introduction as these are already covered in the Methods section. The closing paragraph now concisely states the gap, the study purpose, and the scope of the protocol.

> **Comment:** *Some methodological sections could be streamlined for clarity.*

Response: The Planned statistical analysis section has been restructured to map directly to the three study objectives ("For the first objective...", "For the second objective...", "For the third objective..."), improving clarity and readability.

> **Comment:** *Ensure all abbreviations (e.g., UPRN, BLPU) are clearly defined at first use.*

Response: We have verified that all abbreviations, including UPRN (Unique Property Reference Number), BLPU (Basic Land and Property Unit), CHI (Community Health Index), PHS (Public Health Scotland), SIMD (Scottish Index of Multiple Deprivation), RERI (Relative Excess Risk due to Interaction), and eDRIS (Electronic Data Research and Innovation Service), are defined at first use.

> **Comment:** *Figures (especially Figure 2) could be improved in readability.*

Response: Both figures have been substantially revised. Figure 1 has been redrawn for improved clarity, showing the data linkage steps, data sources, eligibility criteria, and cohort definition in a clear sequential flow. Figure 2 (DAG) has been revised to distinguish confounding pathways (solid arrows) from potential mediating pathways (dashed arrows), the label "Health & Risk Confounders" has been changed to "Health-related covariates," "Household Type" has been updated to "Residential Setting," and multigenerational household status has been repositioned to the sociodemographic confounders box. The figure legend has been updated to explain these visual conventions.

Additional Changes

In addition to addressing the editor and reviewer comments, the following changes were made to strengthen the manuscript:

• The term "household type" has been replaced with "residential setting" throughout the manuscript for greater precision and consistency.

• The disability definition reference has been updated from the UK government learning disability page to the WHO International Classification of Diseases, 11th Revision (ICD-11) definition of disorders of intellectual development, and the text now specifies the "Scottish Census context" to differentiate from the English/Welsh Census approach.

• The abstract Methods and analysis section has been revised to more accurately reflect the data linkage architecture, the use of BLPU codes for residential setting classification, and the inclusion of both multiplicative and additive interaction assessment.

• The covariates section heading "Health and risk-related covariates" has been simplified to "Health-related covariates."

• A new WHO ICD-11 reference has been added (reference 34), replacing the previous UK government learning disability reference.

• The funding statement has been updated to include ADR Scotland: 2026–2031 (UKRI3324).

VERSION 2 - REVIEW

**Reviewer**	**1**
**Name**	**Fernández-Lázaro, Diego**
**Affiliation**	**Universidad de Valladolid Facultad de Ciencias**
**Date**	**16-Apr-2026**
**COI**	

Dear Authors,

Thank you for the opportunity to review this well-written protocol examining the interplay between disability, household living arrangements, and COVID-19 outcomes in Scotland .

This is a timely and policy-relevant study, and the use of population-wide linked administrative data is a major strength.

Major comments

The protocol is methodologically strong and clearly structured, but could benefit from greater clarity in some analytical decisions:

The rationale for choosing cause-specific Cox models over competing risk models is appropriate but could be slightly shortened and clarified for readability.

The approach to handling potential misclassification of disability (2011 Census vs 2020 baseline) is well acknowledged, but consider adding more detail on how sensitivity analyses will specifically address this.

The section on effect modification (additive vs multiplicative interaction) is robust but may be overly technical for some readers. Consider simplifying or briefly clarifying interpretation for a broader audience.

The Introduction is strong but somewhat lengthy and repetitive in places (especially towards the end). A modest reduction would improve readability.

Minor comments

Language is generally excellent, but minor editing for conciseness is recommended.

Some sections (e.g., Methods – statistical analysis) could be slightly streamlined without losing rigor.

Ensure full alignment with BMJ Open reporting style (e.g., clarity, accessibility to multidisciplinary readers).

Conclusion

This is a high-quality protocol addressing an important public health question with strong methodological rigor. Only minor clarifications and stylistic improvements are needed.

Recommendation: Minor revision

VERSION 2 - AUTHOR RESPONSE

Reviewer 1 Comments (Dr. Diego Fernández-Lázaro, Universidad de Valladolid)

General assessment: The Reviewer notes that this is a timely and policy-relevant study, that the use of population-wide linked administrative data is a major strength, and that the protocol is methodologically strong and clearly structured. We thank the Reviewer for this positive assessment and for the detailed feedback provided below.

Major Comments

1. Rationale for cause-specific Cox models

Comment: *The rationale for choosing cause-specific Cox models over competing risk models is appropriate but could be slightly shortened and clarified for readability.*

Response: We have revised this section to improve readability. The rationale has been condensed into a single, clearer sentence. The Planned statistical analysis section now reads: "Cause-specific Cox proportional hazards models will be used throughout rather than subdistribution (Fine–Gray) approaches, consistent with the aetiological aim of quantifying how disability and household living arrangements influence the rate at which COVID-19 events occur, rather than predicting cumulative incidence in the presence of competing events." The parenthetical definition of the cause-specific hazard has been removed, and the two previous sentences describing the justification have been merged into one. The limitation regarding cumulative incidence interpretation is retained in the following sentence.

2. Sensitivity analyses for disability misclassification

Comment: *The approach to handling potential misclassification of disability (2011 Census vs 2020 baseline) is well acknowledged, but consider adding more detail on how sensitivity analyses will specifically address this.*

Response: We thank the Reviewer for this suggestion and have considered it carefully. The 2011 Scottish Census is the only population-wide source of self-reported disability available within our linked data, and a contemporaneous 2020 measure against which to directly validate classification is not available within the approved data sources. Formal sensitivity analyses to quantitatively recalibrate the exposure definition are therefore not feasible within the current data permissions. We have, however, strengthened the text in the Disability status subsection to make the planned analytical approach to this limitation explicit. Specifically, an additional sentence has been added to state that "The potential impact of this measurement gap on observed associations for physical disability will be explicitly acknowledged when interpreting the findings." This clarifies that, while formal sensitivity analysis is constrained by data availability, the likely direction of bias (attenuation of physical disability associations towards the null, as already described in the preceding text) will be transparently discussed when presenting results. We have not overstated what can feasibly be delivered at the analysis stage.

3. Effect modification (additive vs multiplicative interaction)

Comment: *The section on effect modification (additive vs multiplicative interaction) is robust but may be overly technical for some readers. Consider simplifying or briefly clarifying interpretation for a broader audience.*

Response: We agree that the section would benefit from added accessibility for a multidisciplinary readership. The technical description has been retained in full for methodological completeness, but a plain-language clarification has been added at the end of the paragraph. The new text reads: "In practical terms, multiplicative interaction indicates whether the combined effect of disability and a given household living arrangement exceeds the product of their separate relative effects, whereas additive interaction indicates whether their combined absolute effect exceeds the sum of their separate effects.

Both scales provide complementary information: multiplicative interaction is the conventional measure reported in epidemiological studies, while additive interaction is particularly informative for public health planning, as it identifies subgroups in which disability and household context together produce disproportionately elevated absolute risk, and therefore where targeted interventions may have the greatest impact." This provides a clearer interpretive bridge for non-specialist readers while preserving the original statistical rigour.

4. Introduction length and repetition

> **Comment:** *The Introduction is strong but somewhat lengthy and repetitive in places (especially towards the end). A modest reduction would improve readability.*

Response: We thank the Reviewer for this helpful observation. The Introduction has been shortened through three targeted deletions, concentrated in the paragraphs flagged as repetitive. First, the transitional opening sentence of the fourth paragraph ("Global research confirms that residential and household environments are central determinants of COVID-19 transmission and outcomes, yet substantial gaps remain") has been deleted, as its content overlaps with the opening of the preceding paragraph. Second, the recap sentence opening the closing paragraph ("In summary, little is known about how residential setting and household size shape COVID-19 risks among disabled populations, in Scotland and internationally") has been removed, as this point is already made in the preceding Scotland-specific paragraph. Third, the generic bridge sentence ("Addressing these gaps is essential for understanding the social patterning of pandemic-related vulnerability and for informing more inclusive public health planning") has also been removed. The Introduction now moves from the Scotland-specific evidence gap directly to a concise statement of the protocol's purpose and its placement within the broader research programme, resulting in a more focused and less repetitive closing.

Minor Comments

5. Language and conciseness

> **Comment:** *Language is generally excellent, but minor editing for conciseness is recommended.*

Response: We have undertaken a focused conciseness pass on the sections most likely to benefit. Specific changes include: (i) the Study design and data sources subsection has been tightened so that the opening sentence now reads "This study will be a retrospective cohort study using linked, routinely collected administrative data covering the majority of the Scottish population," removing repetition with the Aims and objectives section above; (ii) the opening sentence of the Planned statistical analysis section has been revised from "Cox proportional hazards regression will be used as the primary modelling framework to estimate associations between exposures and time-to-event for COVID-19 outcomes" to the more direct "Cox proportional hazards regression will be the primary modelling framework for time-to-event analysis of COVID-19 outcomes"; and (iii) the baseline characteristics sentences have been streamlined to reduce repetition.

6. Methods – statistical analysis streamlining

> **Comment:** *Some sections (e.g., Methods – statistical analysis) could be slightly streamlined without losing rigour.*

Response: In addition to the Cox rationale simplification described under Major Comment 1, the statistical analysis section has been further streamlined as noted above (opening sentence rewording, outcomes list tightened to "infection, hospitalisation, mortality," and baseline characteristics sentences de-duplicated). The full technical detail of the sequential adjustment strategy, interaction analyses, and sensitivity analyses has been preserved to maintain methodological rigour.

7. BMJ Open reporting style

Comment: *Ensure full alignment with BMJ Open reporting style (e.g., clarity, accessibility to multidisciplinary readers).*

Response: We have attended to this in two specific ways. First, the plain-language clarification added to the effect modification paragraph (see Major Comment 3 above) improves accessibility for multidisciplinary readers. Second, the conciseness edits described above reduce technical density without sacrificing content. The manuscript continues to follow the RECORD and STROBE reporting frameworks for studies using routinely collected health data, consistent with BMJ Open requirements for protocol manuscripts.
